# Mitochondrial-derived peptides, HNG and SHLP3, protect cochlear hair cells against gentamicin

**DOI:** 10.1038/s41420-024-02215-9

**Published:** 2024-10-21

**Authors:** Yu Lu, Ewelina M. Bartoszek, Maurizio Cortada, Daniel Bodmer, Soledad Levano Huaman

**Affiliations:** 1 Department of Biomedicine, University of Basel Hospital, Basel, Switzerland; 2 Department of Otolaryngology, Head and Neck Surgery, University of Basel Hospital, Basel, Switzerland

**Keywords:** Recombinant peptide therapy, Drug development, Hair cell

## Abstract

Preservation of hair cells is critical for maintaining hearing function, as damage to sensory cells potentially leads to irreparable sensorineural hearing loss. Hair cell loss is often associated with inflammation and oxidative stress. One promising class of bioactive peptides is mitochondrial-derived peptides (MDPs), which have already been proven to protect various tissues from cellular stresses and delay aging processes. Humanin (HN) is one of the best-known members of this family, and recently, we have shown its protective effect in hair cells. The synthetic derivate HN S14G (HNG) has a more potent protective effect than natural HN making it a more useful peptide candidate to promote cytoprotection. A less-known MDP is small humanin-like peptide 3 (SHLP3), which has cytoprotective effects similar to HN, but likely acts through different signaling pathways. Therefore, we examined the effect of exogenous HNG and SHLP3 in auditory hair cells and investigated the molecular mechanisms involved. For this purpose, explants of the organ of Corti (OC) were treated with gentamicin in the presence and absence of HNG or SHLP3. Administration of HNG and SHLP3 reduced gentamicin-induced hair cell loss. The protective mechanisms of HNG and SHLP3 in OC explants included, in part, modulation of AKT and AMPKα. In addition, treatment with HNG and SHLP3 reduced gentamicin-induced oxidative stress and inflammatory gene overexpression. Overall, our data show that HNG and SHLP3 protect hair cells from gentamicin-induced toxicity. This offers new perspectives for the development of therapeutic strategies with MDPs against hearing loss.

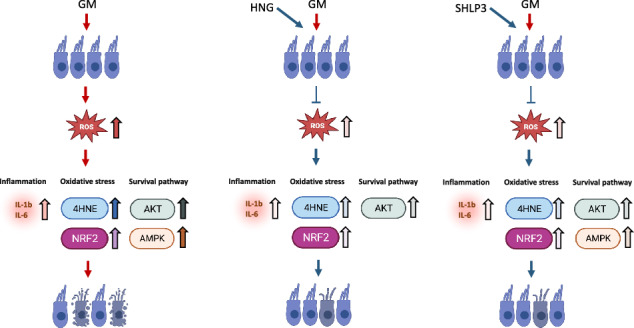

## Introduction

The auditory hair cells are essential for the perception of sound. They convert the mechanical sound waves into electrical impulses, which are transmitted to the brain via afferent synapses of auditory nerve cells. Loss of hair cells often results in permanent sensorineural hearing loss. Aminoglycosides, noise exposure, infections, chemotherapeutic agents, aging, and genetic conditions are factors that damage auditory hair cells [1]. The aminoglycoside gentamicin selectively damages sensory hair cells by triggering a series of intracellular events, including disruption of reactive oxygen species (ROS) balance and dysregulation of protein homeostasis [2–5]. Indeed, the high production of ROS in the cochlear cells is believed to be caused by gentamicin impairing the bioenergetic function of the mitochondria and reducing nicotinamide adenine dinucleotide [6]. Gentamicin also induces mitochondrial ribosome dysfunction, leading to inhibition of protein synthesis [5]. Inflammatory responses of cochlear cells were also observed after gentamicin exposure [7, 8].

Strategies to protect sensory hair cells rely on attenuating damaging effects by inhibiting one or more steps of ototoxic mechanisms. Several compounds have been proposed to protect sensory hair cells, but the development of an effective therapy to prevent cell loss or rescue damaged cells remains an ongoing challenge. Over the past decade, interest in therapeutic peptides has increased as technologies for design and production have improved, and new routes of delivery have emerged [9]. In fact, one class of bioactive molecules with cytoprotective properties is mitochondrial-derived peptides (MDPs) [10, 11]. MDPs are encoded by small open reading frames in the mitochondrial genome and are essential for maintaining mitochondrial bioenergetic activity [12]. Humanin (HN), which is encoded in the 16S region of the mitochondrial genome and translated into a 24-amino acid peptide, was the first MDP to be discovered. A synthetic derivative of HN, in which glycine in position 14 was replaced by serine (HNG), displayed 1000-fold higher efficacy and protective properties already in the nanomolar range [13]. Other members of this MDP family encoded in 16S region are small humanin-like peptides, SHLP1-6. SHLP3 is less studied, but it is known to have similar protective effects as HN [10]. HN is expressed in several organs such as the brain, heart, kidney, liver, testis, and skeletal muscle. HN as a secreted peptide was detected in blood circulation and is thought to be transported to target tissues [14]. HN appears to act via both intrinsic and extrinsic signaling pathways. Several HN-interacting proteins were described, including the insulin-like growth factor-binding protein-3 (IGFBP-3) and Bcl2-associated X protein (BAX) [15, 16]. HN acts extrinsically as a ligand for cell surface receptors such as formyl peptide receptor-like 1 (FPRL1) and 2 (FPRL2) as well as the trimeric cytokine receptor consisting of ciliary neurotrophic factor receptor alpha (CNTFRα), WSX1, and glycoprotein 130 (gp130) [17, 18].

Cytoprotective properties of MDPs have been demonstrated in various tissues and recently also in the inner ear, where we investigated the effect of HN and MOTS-c against gentamicin [19]. In light of these findings, there is great interest in investigating the mechanisms of other bioactive peptides in auditory hair cells under normal and pathological conditions. Thus, in the present study, HNG and SHLP3 were administrated in an ototoxic model in-vitro. We found HNG and SHLP3 protected hair cells from the adverse effects of gentamicin. We further identified their possible mechanisms of action in hair cells, including potential signaling targets.

## Results

### HNG and SHLP3 protect rat cochlear hair cells against gentamicin-induced apoptosis

**Fig. 1:**
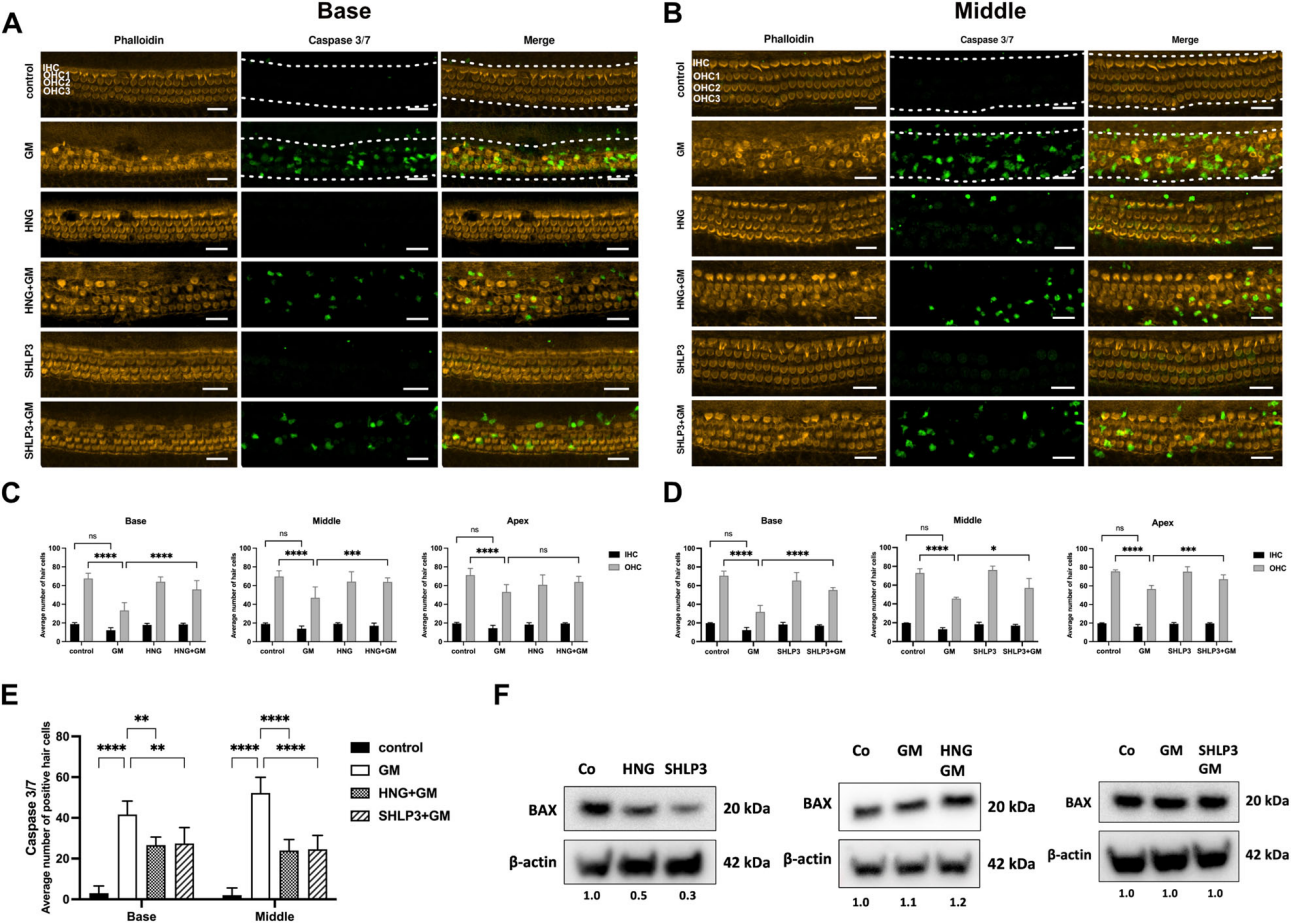
HNG and SHLP3 protect rat cochlear hair cells against gentamicin-induced damage. Representative images of phalloidin-stained hair cells (orange) and caspase 3/7-positive hair cells (green) from the basal (**A**) and middle (**B**) turns of the cochlea. **C**, **D** Quantification of inner hair cell (IHC) and outer hair cell (OHC) survival in basal, middle and apical regions. **E** Quantification of caspase 3/7-positive hair cells in the basal and middle regions. Explants were treated for 24 h with 200 μM gentamicin (GM) in combination with or without 1 µM humanin S14G (HNG) or 0.08 µM SHLP3. GM reduced the number of surviving hair cells, which was increased in the presence of HNG and SHLP3. At least five explants for each condition were used. All images are maximum-intensity projections of a stack of optical sections. Examples of regions used to measure immunofluorescence intensities are indicated by white dashed lines. Scale bar = 20 μm. Values are presented as mean+S.D. ****P < 0.0001, ***P < 0.001, **P < 0.01, *P < 0.05, ns no significant. **F** Western blot results showing the expression of BAX after 24 h treatment with GM with or without HNG treatment. The bands of β-actin from SHLP3-treated samples are the same as in Fig. 7A (for 4-HNE). At least six explants were used as a sample pool for each condition.

We previously showed that 10 µM HN protects auditory cells from gentamicin [19]. Here, we tested HN’s more potent derivative at a lower concentration, 1 μM HNG. At this concentration, HNG was not toxic to hair cells (Fig. 1A–C). SHLP3 was reported previously to exert a protective effect at a concentration of 0.1 μM [10]. We investigated different concentrations of SHLP3 between 0.04 and 0.4 µM (Supplementary Fig. S1). All these concentrations were non-toxic to hair cells. We used the concentration of 0.08 µM for hair cell damage and Western blot experiments and increased it to 0.1 µM for real-time PCR and immunofluorescence experiments (Fig. 1A, B, D) to obtain more robust results.

To test whether exogenous HNG and SHLP3 protect hair cells from ototoxicity, OC explants were pretreated with the MDPs and then exposed to 200 µM gentamicin in the presence or absence of the MDPs. Outer hair cells were previously shown to be lost by ~50% at this gentamicin concentration [19]. As expected, gentamicin exposure significantly reduced the outer hair cell numbers in OC explants compared to untreated samples (Fig. 1A–D). Administration of HNG or SHLP3 to gentamicin-exposed explants significantly reduced outer hair cell loss in OC explants. The inner hair cells were not affected by this gentamicin concentration, and no significant loss of inner hair cells was observed. Consequently, the observed protective effect of the peptides concerned the outer hair cells (Fig. 1A–D). However, the protective effect of the peptides on the inner hair cells could also occur under conditions detrimental to these cells. This result demonstrates the protective role of MDPs for hair cells against gentamicin-induced toxicity in rat cochlea.

To verify whether the loss of hair cells was attributable to apoptosis, the explants were stained with caspase 3 and 7. In such immunofluorescence experiments, we focus on the medial and basal regions, because the apical region of neonatal rats was not affected by exposure to 200 µM gentamicin. At the same time, we are aware that the effect of SHLP3 on the apical region will also not be examined by immunofluorescence. To allow adequate analysis of caspase-positive cells in all experimental groups, we set the area for quantification of immunofluorescence intensities to include both inner and outer hair cells, as apoptotic outer hair cells lose their position and are found in the area of inner hair cell (IHC). Few apoptotic IHC are also counted, but this does not affect the quantification as the number of IHC was not significantly different between the groups. In this assay, untreated explants showed almost no positive hair cells for caspase 3/7. A significantly high number of positive hair cells was observed in gentamicin-exposed explants, but this number was reduced in explants exposed to gentamicin together with MDPs (Fig. 1E). The data suggest that MDP treatments may protect hair cells from caspase-mediated damage by gentamicin.

We analyzed a key pro-apoptotic protein, BAX, reported to bind HN and decrease in expression after HNG administration [16, 20]. As expected, BAX total protein expression was reduced by the addition of HNG to OC explants. Similarly, SHLP3 attenuated BAX expression (Fig. 1F and Supplementary Fig. S3). On the other hand, BAX expression was not decreased when OC explants were exposed to gentamicin together with HNG or SHLP3 compared to explants exposed to gentamicin alone.

### HNG decreases *Rattin* expression in gentamicin-exposed hair cells

**Fig. 2:**
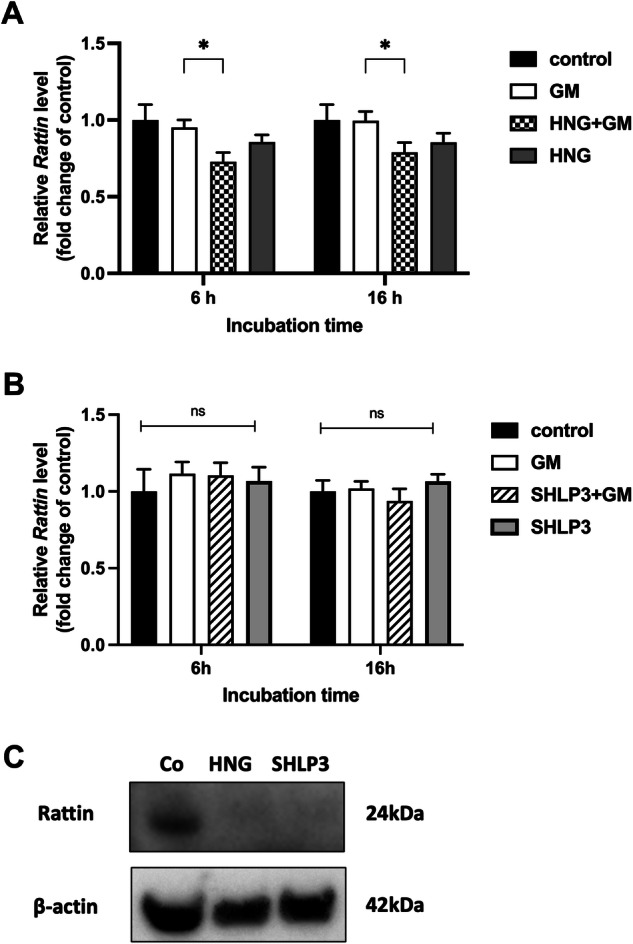
HNG decreases *Rattin* expression in gentamicin-exposed cochlear hair cells. The expression of *Rattin* in OC explants was measured by real-time PCR. OC explants were treated with 200 μM gentamicin (GM) in combination with or without 1 µM HNG (**A**) or 0.1 µM SHLP3 (**B**) for 6 h and 16 h. At least three explants were used as sample pool for each condition. Values are presented as mean+S.D. *P < 0.05, ns no significant between all groups. **C** Western blot results showing the expression of RATTIN after 24-h peptide treatment (1 µM HNG or 0.08 µM SHLP3). At least six explants were used as a sample pool for each condition.

*Rattin* gene encodes a rat peptide that is homologous to HN. We investigated its expression at the RNA and protein levels. *Rattin* transcript levels in OC explants remain unchanged after exposure to gentamicin alone or HNG alone. Administration of HNG to explants exposed to gentamicin resulted in a decrease in *Rattin* transcripts compared to explants exposed to gentamicin alone. This effect was observed at both time points, 6 h and 16 h (Fig. 2A). SHLP3, in contrast, did not affect the transcript levels of *Rattin* under any conditions (Fig. 2B). At the protein level, RATTIN was undetectable in MDP-exposed explants, but present in control explants (Fig. 2C and Supplementary Fig. S4). These data indicate that in the presence of HNG, the transcription of *Rattin* is attenuated. Furthermore, HNG and SHLP3 restricted the production of RATTIN.

### Exogenous HNG and SHLP3 are localized intracellularly and on the cell surface of hair cells

**Fig. 3:**
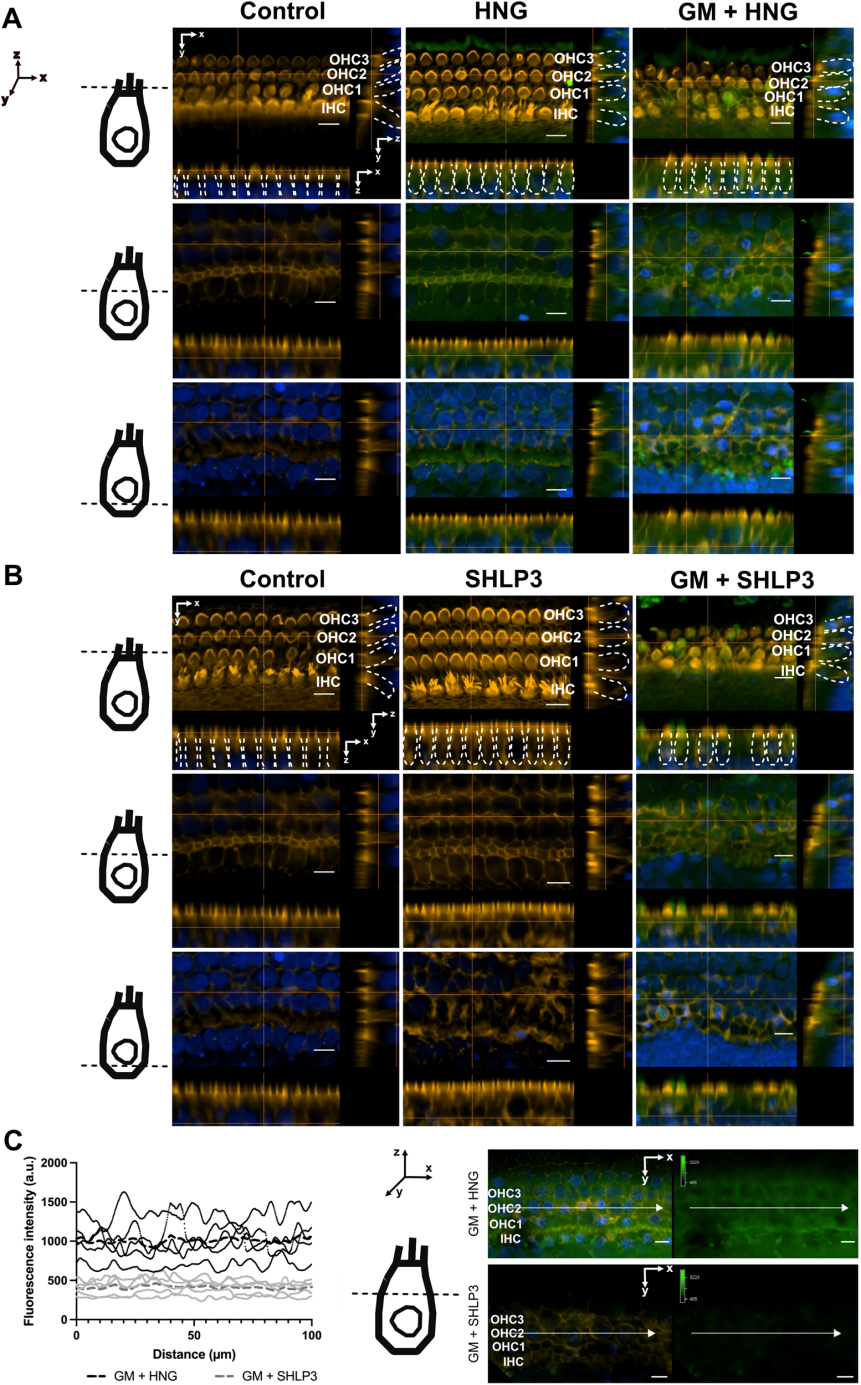
Exogenous HNG and SHLP3 are localized intracellularly and on the cell surface of hair cells when exposed to gentamicin. Representative images of FITC-HNG (**A**, green) and FITC-SHLP3 (**B**, green) in the middle turns of the cochlea from individual slices of a Z-stack. To better visualize the peptides, different fluorescence intensities were used for FITC-HNG and FITC-SHLP3, while maintaining the settings within each peptide group. The schematic representation of the hair cell (HC) indicates the different localization of the orthogonal views. The inner (IHC) and outer hair cells (OHC) are marked by dashed white lines. **C** Fluorescence intensity profiles of FITC-HNG or FITC-SHLP3 from a single XY plane image of a Z-stack. The same fluorescence intensity settings were used for both labeled peptides. Each curve with black (FITC-HNG) or gray (FITC-SHLP3) dots corresponds to a sample representing a randomly selected middle section (100 µm). The dashed lines refer to the average values of all 4 samples. The schematic representation of the hair cell (HC) shows the position of the individual image in the XY plane. In the representative image of a single XY plane of a Z stack, the white arrows indicate the position of the measurement of the fluorescence intensities. Explants were treated with 200 μM gentamicin (GM) in combination with 1 µM HNG and 0.1 µM SHLP3. Hair cells (orange) and nuclei (blue) were stained with phalloidin and DAPI, respectively. *n* = 4. Scale bar = 10 µm.

It was found that exogenous HNG is taken up by retinal pigment epithelium (RPE) cells [21]. Therefore, we investigated whether the exogenous HNG and SHLP3 are taken up into the hair cells or remain on the surface of the hair cells. For this purpose, labeled FITC-HNG and FITC-SHLP3 were added to OC explants alone or together with gentamicin. When the explants were incubated with FITC-labeled peptides alone, FITC labeling was found mainly on the cell surface near the stereocilia region of the hair cells (Fig. 3A, B), and only small amounts of FITC-HNG were detected in the cell bodies (Fig. 3A). When gentamicin was simultaneously administered to the explants, FITC labeling was detected on the surface and in the bodies of the hair cells (Fig. 3A, B, Supplementary videos 1–2). FITC labeling on the surface of the hair cells was observed as strong green immunofluorescence on the upper surface of some hair cells when the images were taken on the plane of the stereocilia. FITC labeling in the bodies of almost all hair cells was observed in the planar images along the *z*-axis. Representative single planes above and below the nuclei are shown in Fig. 3A, B. FITC labeling of HNG and SHLP3 showed different fluorescence intensities in the explant samples, therefore the imaging parameters for the experimental samples of each peptide were adjusted accordingly (Fig. 3A, B). To compare the differences in labeling intensity between FITC-HNG and FITC-SHLP3, the explants were imaged with the same acquisition parameters and fluorescence intensity profiles of HNG and SHLP3 were generated. A twofold stronger labeling of FITC-HNG than of FITC-SHLP3 was observed in the organs (Fig. 3C), which may be due to the different concentrations administered. Labeled HNG peptides were also identified in the support cells, as shown in the orthogonal views (below the HC nucleus, Fig. 3A) and the YZ-projection images (Supplementary Fig. S2). These data indicate that MDPs mainly bind to the surface of hair cells and enter the cells through the damaged cell membrane.

### MDPs neither activate STAT3 nor ERK1/2 in gentamicin-exposed explants

**Fig. 4:**
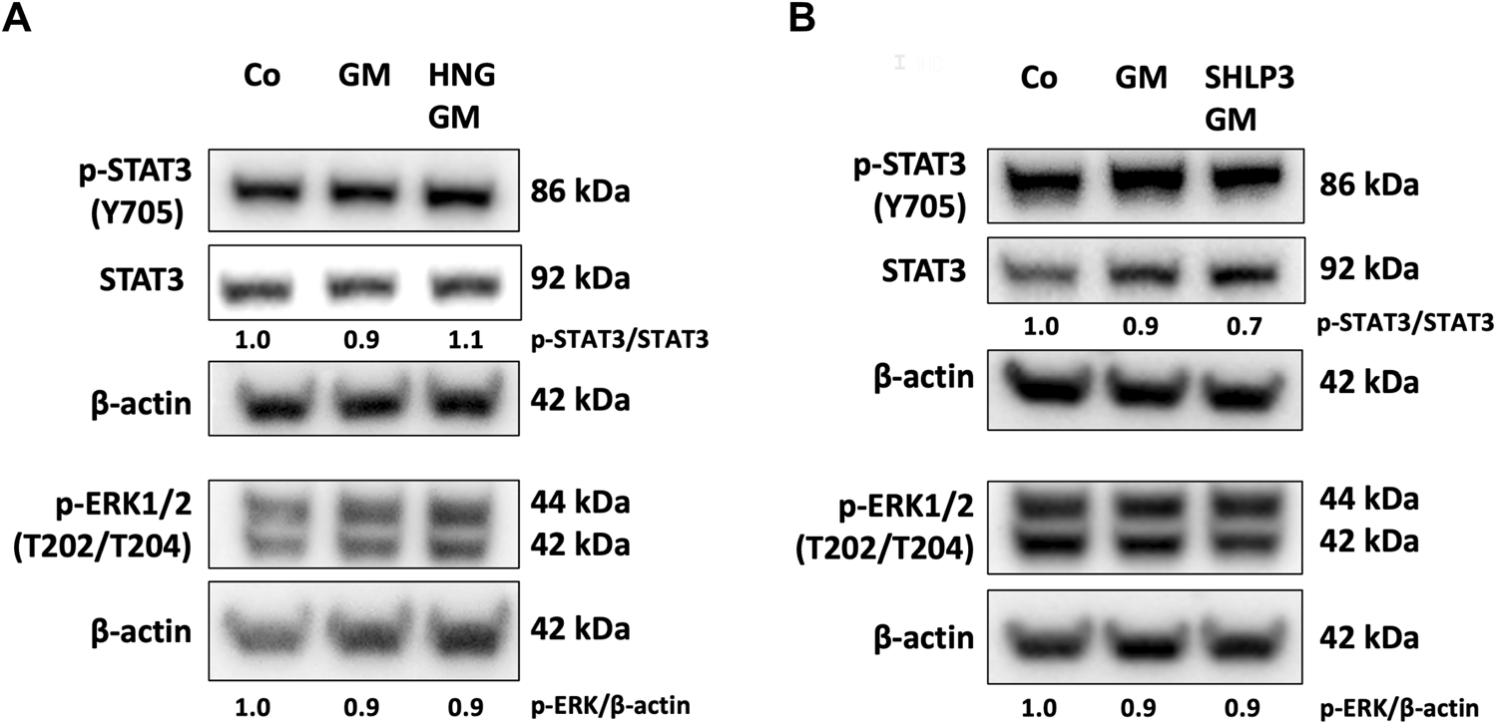
MDPs neither activate STAT3 nor ERK1/2 in gentamicin-exposed explants. Western blot results showing the detection of STAT3 and ERK1/2 after 24 h treatment with 200 μM gentamicin (GM) in combination with or without 1 µM HNG (**A**) or 0.08 µM SHLP3 (**B**). At least six explants were used as a sample pool for each condition. Densitometric analysis of each protein normalized against to control (Co) is shown. The bands of β-actin from (**A**, lower membrane) are the same as in Fig. 7A. The bands of β-actin from (**B**) are the same as in Fig. 6A. The membranes were stripped and re-probed again as described in the material and methods section.

HN has been reported to act via activation of STAT3 and ERK1/2 [22, 23]. Like HNG, SHLP3 also activates ERK1/2 [10]. However, such activation of STAT3 has not always been demonstrated [19]. To investigate the possible activation of STAT3 and ERK1/2, HNG and SHLP3 were added to OC explants exposed to gentamicin. HNG and SHLP3 treatments did not cause significant changes in the phosphorylation of STAT3 and ERK1/2 (Fig. 4A, B and Supplementary Fig. S5). These results suggest that HNG and SHLP3 may not act via activation of STAT3 and ERK1/2 in OC explants.

### HNG decreases gentamicin-induced activation of AKT in cochlear cells

**Fig. 5:**
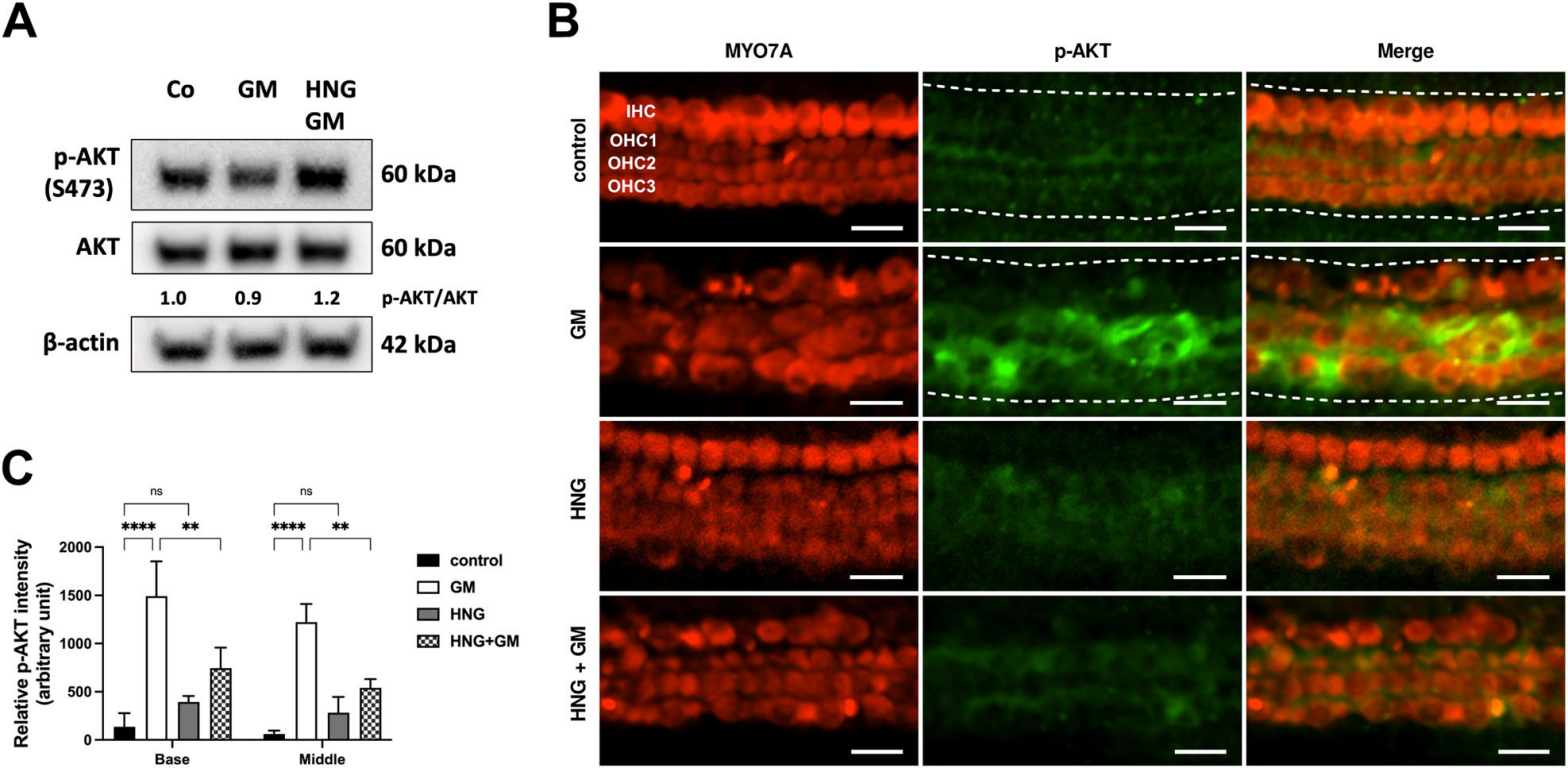
The protective effect of HNG is associated with phosphorylation of AKT in cochlear cells. **A** Western blot results showing the detection of p-AKT after 24 h treatment with gentamicin (GM) and 1 µM HNG. At least six explants were used as the sample pool for each condition. Densitometric analysis of each protein normalized against to control (Co) is shown. **B** Immunofluorescence staining of myosin7a (MYO7A) and p-AKT in OC explants (middle turn) after GM and 1 µM HNG treatment for 20 h. Examples of sections used to measure immunofluorescence intensities are indicated by a white dashed line. **C** Quantification of p-AKT intensity for each condition. *n* = 3. Scale bar = 20 μm. Values are presented as mean+S.D. ****P < 0.0001, **P < 0.01, ns not significant.

In addition to the known STAT3 and ERK1/2 signaling pathways, HN also activates the AKT signaling pathway [19, 23, 24]. We, therefore, investigated the protein expression of AKT and its phosphorylated form in gentamicin-exposed OC explants in the presence of HNG. In protein lysates from OC explants, we found that p-AKT-S473 was not altered in gentamicin-HNG-exposed explants compared to only gentamicin-exposed explants (Fig. 5A and Supplementary Fig. S6). Whereas examination of the OC explants by immunofluorescence revealed that the immunofluorescence signal of p-AKT-S473 was significantly increased in gentamicin-exposed explants and reduced in the presence of HNG together with gentamicin (Fig. 5B, C). Furthermore, HNG alone showed no effect on basal p-AKT-S473. These results suggest that HNG exerts its protective effect in hair cells against gentamicin via the modulation of AKT.

### SHLP3 decreases gentamicin-induced activation of AKT and AMPKα in cochlear cells

**Fig. 6:**
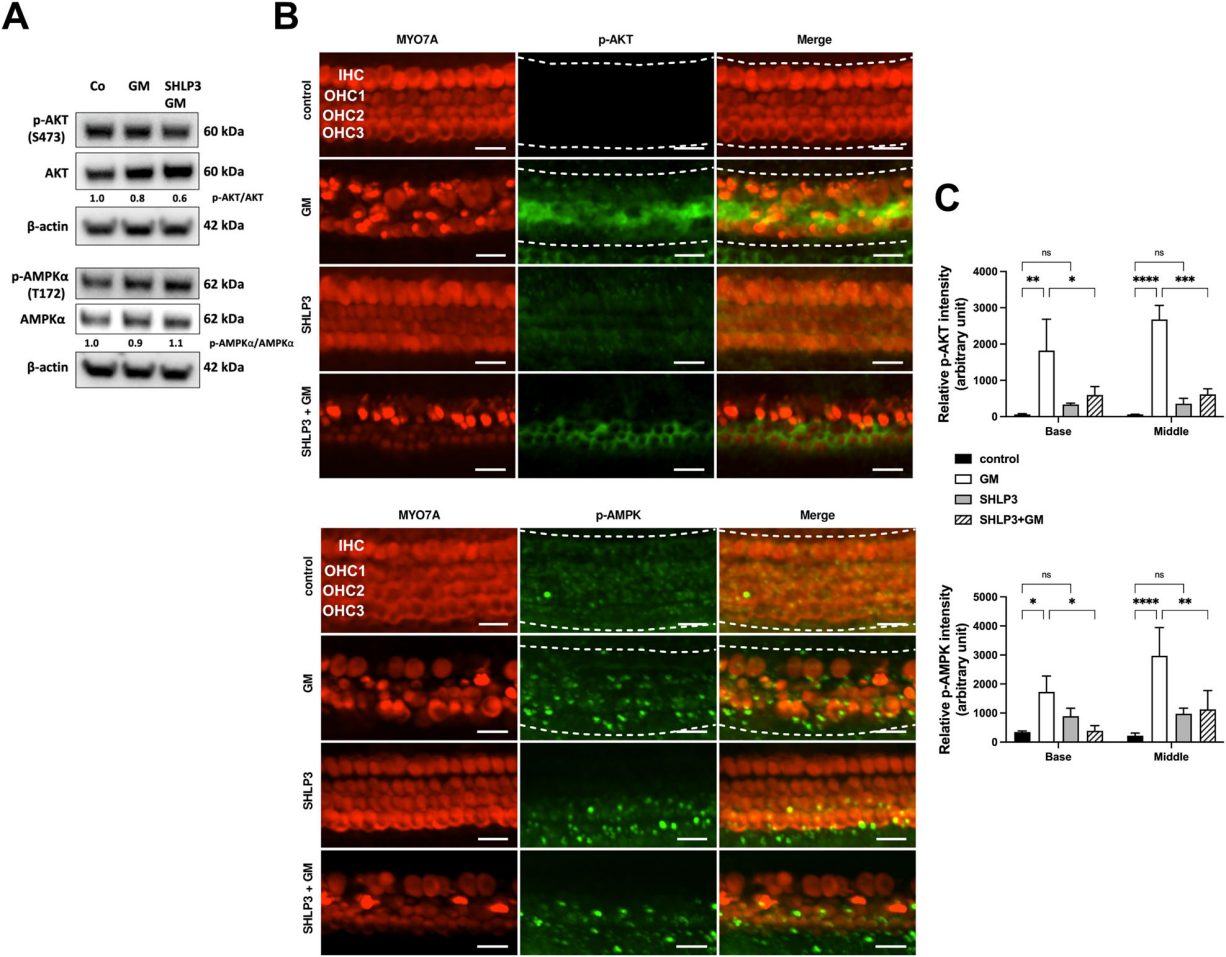
The protective effect of SHLP3 is associated with phosphorylation of AKT and AMPKα in cochlear hair cells. **A** Western blot results show the expression of p-AKT and p-AMPKα after gentamicin (GM) and 0.08 µM SHLP3 treatment for 24 h. At least six explants as a sample pool of each condition were used. Densitometric analysis of each protein normalized against to control (Co) is shown. **B** Immunofluorescence staining of myosin7a (MYO7A), p-AKT and p-AMPKα in OC explants (middle turn) after GM and 0.1 µM SHLP3 treatment for 20 h. Examples of sections used to measure immunofluorescence intensities are indicated by a white dashed line. **C** Quantification of the p-AKT and p-AMPKα intensities for each condition. *n* = 3. Scale bar = 20 μm. Values are presented as mean+S.D. ****P < 0.0001, ***P < 0.001, **P < 0.01, *P < 0.05, ns no significant.

The mechanism by which SHLP3 exerts cytoprotective effects is largely unknown, but it was hypothesized to function similarly to HN. We, therefore, investigated the protein expressions of AKT and AMPKα along with their phosphorylated forms. In protein lysates from OC explants, we found a slightly increased phosphorylation of AMPKα in gentamicin-SHLP3-exposed explants compared to gentamicin-exposed explants alone (Fig. 6A and Supplementary Fig. S7). In contrast, p-AKT-S473 remained almost unchanged under all conditions (Fig. 6A). On the other hand, examination of the OC explants by immunofluorescence revealed that the immunofluorescence signals of p-AKT-S473 and p-AMPKα-172 were significantly increased in both turns of gentamicin-exposed explants compared to the control explants (Fig. 6B, C). Although the fluorescence intensities were slightly higher in the middle turn than in the basal turn, this was not significant. The addition of SHLP3 to gentamicin-exposed explants resulted in a significant decrease in the immunofluorescence signals of p-AKT-S473 and p-AMPKα-172 in both cochlear turns. Furthermore, SHLP3 had no significant effect on the basal levels of p-AKT-S473 and p-AMPKα-172 compared to control samples. These results suggest that SHLP3 exerts its protective effect in hair cells against gentamicin via modulation of AKT and AMPKα.

### MDPs decrease gentamicin-induced oxidative stress in hair cells

**Fig. 7:**
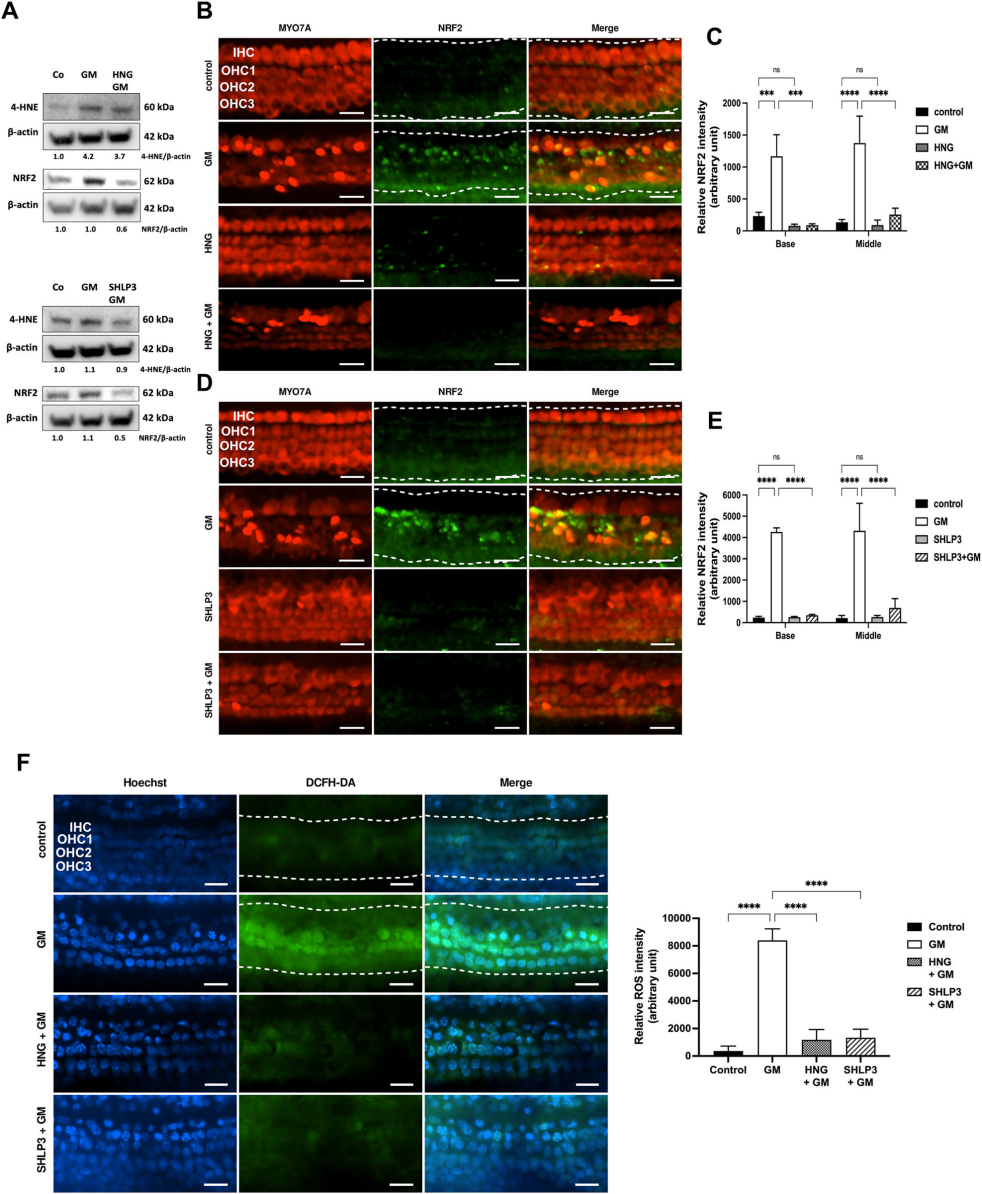
HNG and SHLP3 prevent gentamicin-induced hair cell damage by reducing oxidative stress in OC explants. **A** Western blot results showing the expression of 4-HNE and NRF2 after 24 h treatment with gentamicin (GM) and 1 µM HNG or 0.08 µM SHLP3. At least six explants were used as a sample pool for each condition. Densitometric analysis of each protein normalized against to control (Co) is shown. **B**, **C** Immunofluorescence staining of myosin7a (MYO7A) and NRF2 in OC explants (middle turn) after treatment with GM and 1 µM HNG or 0.1 µM SHLP3 for 20 h. *n* = 3. **D**, **E** Quantitative analysis of NRF2 data. **F** HNG and SHLP3 attenuate gentamicin-induced ROS formation. Representative fluorescence images and quantitative analysis of DCFH-DA (indicator for ROS) in cochlear hair cells in the middle turn after different treatments. *n* = 5. Scale bars: 20 µm. Examples of sections used to measure immunofluorescence intensities are indicated by a white dashed line. Different *Y*-axis scales were used for (**C**), (**E**), and (**F**). Values are presented as mean+S.D, ****P < 0.0001, ***P < 0.001, ns no significant.

Gentamicin damages hair cells by increasing ROS levels, thereby inducing oxidative stress [6]. In addition, HNG and SHLP3 exert protective effects by reducing ROS production [10, 21]. To determine whether MDP protection against gentamicin-induced hair cell damage is related to oxidative stress, we examined two key proteins, 4-hydroxynonenal (4-HNE) and nuclear factor erythroid 2-related factor 2 (NRF2). In protein lysates from OC explants, the phosphorylations of 4-HNE and NRF2 were decreased in gentamicin-HNG-exposed explants compared to gentamicin-exposed explants alone (Fig. 7A upper part and Supplementary Fig. S8). While phosphorylation of NRF2 was decreased in gentamicin-SHLP3-exposed explants compared to gentamicin-exposed explants alone (Fig. 7A lower part and Supplementary Fig. S8). Examination of the OC explants by immunofluorescence showed that NRF2 immunofluorescence was significantly higher in gentamicin-exposed explants than in control explants for both cochlear turns (Fig. 7B–E). The addition of HNG or SHLP3 to gentamicin-exposed explants caused a significant decrease in NRF2 immunofluorescence signals in both cochlear turns. Furthermore, neither HNG nor SHLP3 had any effect on the basal level of NRF2. Consistent with our initial experiments (Fig. 1), many MYO7A-labeled hair cells were lost when exposed to gentamicin, and the addition of HNG or SHLP3 attenuated this loss of hair cells (Fig. 7B, D). In the context of oxidative stress, we noted an expected increase in ROS levels in the explants exposed to gentamicin (Fig. 7F). The addition of HNG or SHLP3 to gentamicin-exposed explants resulted in a significant decrease in ROS concentration, with levels comparable to the control samples.

### MDP protection against gentamicin-induced hair cell damage is associated with inflammation

**Fig. 8:**
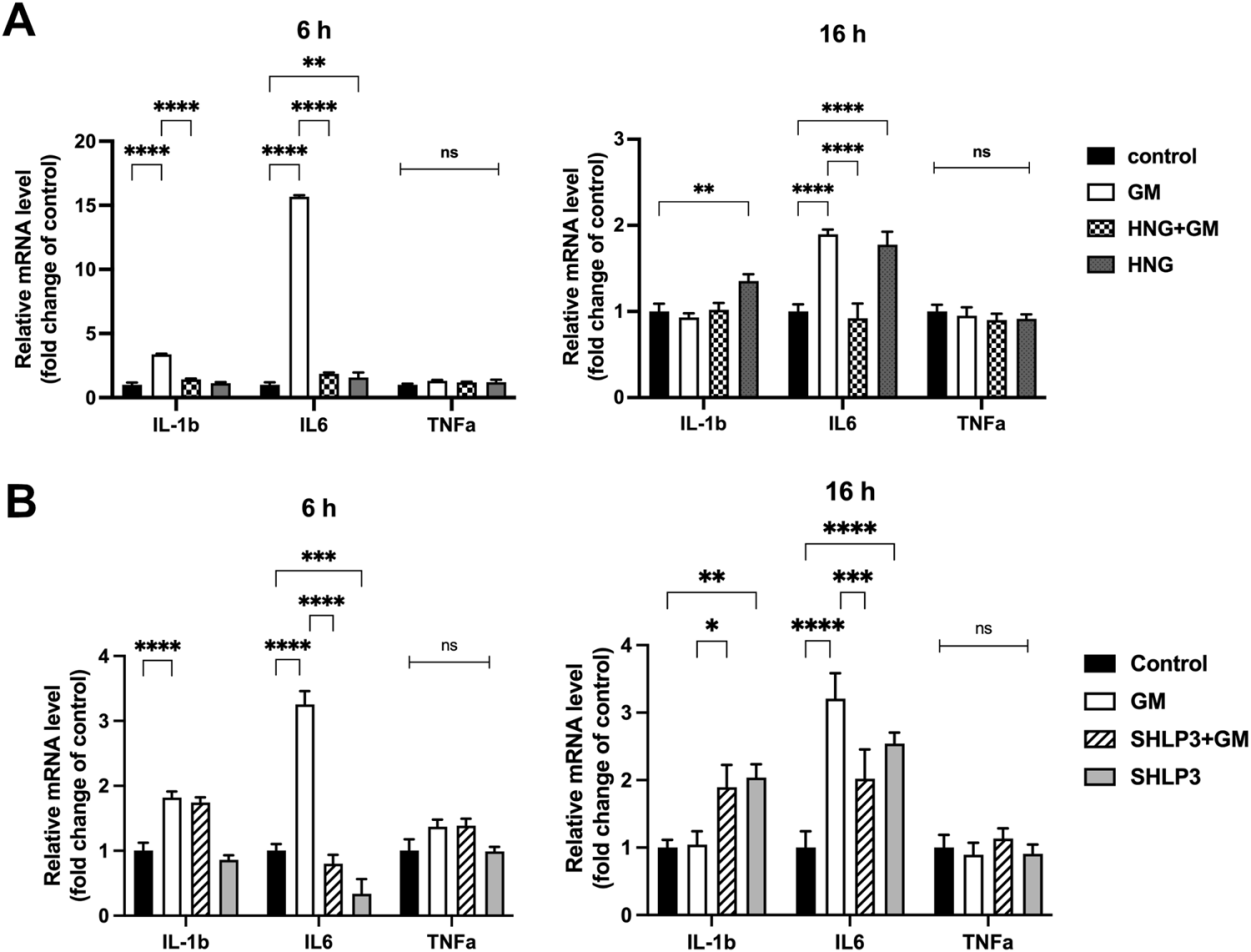
HNG and SHLP3 protection against gentamicin-induced hair cell damage is associated with inflammation. The expression of inflammatory genes in OC explants was measured by real-time PCR. OC explants were treated for 6 h and 16 h with 200 μM gentamicin (GM) in combination with or without 1 µM HNG (**A**) or 0.1 µM SHLP3 (**B**), using at least three explants as a sample pool for each condition. Values are given as mean+S.D. ****P < 0.0001, ***P < 0.001, **P < 0.01, *P < 0.05, ns no significant.

To determine whether MDP protection against gentamicin-induced hair cell damage is related to inflammation, we examined the gene expression of the proinflammatory cytokines *IL-1b*, *IL-6*, and *TNFα*. The expression of *IL-1b* was increased in the presence of gentamicin after 6 h and the addition of HNG, but not SHLP3, decreased its expression to levels comparable to those of control samples (Fig. 8). At 16 h, the expression of *IL-1b* was not affected by gentamicin, but the addition of SHLP3 together with gentamicin increased its expression (Fig. 8B). The expression of *IL-6* was increased in the presence of gentamicin at both time points examined, and the addition of HNG or SHLP3 decreased its expression to levels comparable to those of the control samples (Fig. 8). Although the experiments were performed under identical conditions, the changes in *IL-6* attributable to gentamicin addition varied between the peptide experiments, which could be due to natural biological variation. In addition to these observations, the expression of inflammatory genes was affected by incubation with peptides alone and independently of gentamicin. This effect was most evident after 16 h, where the expression of *IL-1b* and *IL-6* was significantly increased compared to control samples. The levels of *TNFα* were not affected under any condition. This data suggests that MDPs act in part by regulating the expression of inflammatory genes, primarily *IL-1b* and *IL-*6.

## Discussion

We demonstrate that exogenous HNG and SHLP3 have protective properties against gentamicin-induced hair cell loss. HNG attenuated the damaging effects of gentamicin by modulating AKT phosphorylation and SHLP3 by modulating AKT and AMPKα phosphorylation. Furthermore, the protective mechanisms of MDPs against gentamicin were associated with attenuation of oxidative stress and inflammation.

We found that HNG protected hair cells from gentamicin, which is consistent with our previous report, where HN protected hair cells from gentamicin [19]. Similarly, HNG protected neuroblastoma cells SH-SY5Y from oxygen-glucose deprivation (OGD) and reoxygenation [25]. Even micromolar concentrations of HNG protected brain endothelial cells from OGD insult [26]. On the other hand, SHLP3 administration increased cell viability by reducing apoptosis in pancreatic β-cell line and human prostate cancer cells cultured in serum-free media [10]. Consistent with this, we found that SHLP3 and HNG protected hair cells by decreasing the apoptotic caspases 3/7 in gentamicin-exposed explants. Furthermore, we found that the addition of plain HNG or SHLP3 to OC explants decreased BAX expression, and this result is consistent with other reports finding a decrease in BAX by HN [16, 27]. In contrast, BAX expression was not decreased, when MDP was present in the gentamicin-exposed explants. We suggest that the inhibitory binding of MDPs to BAX may still occur, because the total BAX amount may not need to be altered in the explant lysate. Since caspase 3/7 was decreased, we suggest that HNG and SHLP3 protect hair cells from caspase-induced apoptosis.

Administration of synthetic HN decreased the high levels of endogenous *Rattin* in the cytosolic and mitochondrial fractions during testicular stress, while RATTIN protein was not always detected in the cytosolic fraction [28]. The authors concluded that synthetic HN could take over the function of endogenous RATTIN by binding the BAX protein and no additional increase in endogenous RATTIN would be required. In our study, a decrease in *Rattin* transcripts was observed after the administration of HNG to gentamicin-exposed explants, whereas SHLP3 did not affect them. Basal expression of RATTIN protein was undetectable after administration of HNG and SHLP3. We, therefore, hypothesize that HNG decreases the expression of *Rattin* transcripts and takes over RATTIN function to promote hair cell survival under stress conditions. Furthermore, under normal conditions in the presence of exogenous MDPs, RATTIN is likely suppressed by a self-regulatory mechanism of the cells. Further studies involving the functional blockade of RATTIN are required in order to clarify the takeover function of HNG in the hair cells.

Whether HN acts by binding surface receptors or is recruited into the cells is still controversial. Both possibilities of HN action were reported [17, 21, 29]. We found that hair cells were the main target cells for MDPs. It was observed that both FITC-HNG and FITC-SHLP3 were localized on the surface and inside the hair cells of gentamicin-exposed explants, while almost no labeling was found intracellularly in explants treated with FITC-MDPs alone. Similarly, HNG added alone was unable to cross the cytoplasmic membrane of neuronal cells, suggesting that HNG probably exerts its neuroprotective effect by binding to its surface receptor, as reported in the work of Hashimoto [17, 30]. In contrast, HN was rapidly taken up into RPE cells under normal conditions [21]. Our results may indicate that the cell membrane is slightly damaged at an early stage of gentamicin exposure and becomes permeable to the entry of MDP into hair cells. Therefore, the damaged cell membrane could be the main route for peptide entry into the cells. Another possible mechanism for the entry of small amounts of MDP into the cells could be endocytosis-mediated uptake or uptake by transporters on the surface of the hair cells. Further studies are required to clarify the mechanism of peptide entry into hair cells. Here, we did not investigate and define the subcellular location of MDPs, but we know that HNG colocalizes with mitochondria and lysosomes [21, 29, 31]. The subcellular location of MDPs in hair cells remains undetermined in our study.

HN acts extrinsically by binding to a dimeric or trimeric cytokine receptor complex followed by activation of STAT3 and ERK1/2 [17, 21–23]. In addition, HN triggered the activation of ERK1/2 through the binding of FPRL1 and FPRL2 receptors [18]. SHLP3 activated ERK1/2 but did not affect STAT3 [10]. In our study, HNG and SHLP3 did not trigger activation of STAT3 and ERK1/2. Indeed, this lack of STAT3 activation was reported in our previous report using HN [19]. One possible explanation is that activation of STAT3 was not required because pretreatment with HNG or SHLP3 preconditioned the cells preventing the deleterious effects of gentamicin. In this context, activation of ERK1/2 may also not be required for hair cells to survive gentamicin exposure. Another possible explanation is that the activation was transient and not recognized at the time point studied, as observed in another study with neuroblastoma cells [23]. An alternative explanation is the existence of other, previously unknown cell surface receptors since the labeled MDPs were detected on the hair cell surface, suggesting a ligand for cell surface receptors to trigger intracellular responses. Further studies are needed to clarify whether HNG and SHLP bind to other hair cell surface receptors.

The Western blot results did not always provide clear changes in protein expression, so the inclusion of the immunofluorescence method improved the analysis of our data. Since the protein lysate is a pool of all cells contained in the organ of Corti and hair cells comprise less than 10% of all cells in the organ of Corti, the changes in protein expression of hair cells may be diluted and may appear unaltered. In comparison, we were able to localize the target proteins in the hair cells using the immunofluorescence method and thus detect their local changes.

To further characterize the protective mechanism of MDPs, we investigated AKT as a known survival protein that has been already reported to be activated by HN [19, 23, 24]. Our data confirmed that HNG protected hair cells from gentamicin in part by modulation of AKT. Since the mechanism of action of SHLP3 should be similar to that of HN, we included AKT and AMPKα to investigate SHLP3’s protective effect. Like HNG, SHLP3 also modulated the activation of AKT in OC explants. In addition, we found that AMPKα is involved in the protective mechanism of SHLP3. No association between SHLP3 and AMPKα has been reported so far, but similar effects of HN on phosphorylation of AMPKα were found in an in-vivo mouse model of myocardial infarction [27]. Because AMPKα was previously identified as being involved in hair cell survival [32], modulation of AMPKα may be part of the survival mechanism of SHLP3 against gentamicin.

We found that in the presence of MDPs, gentamicin-induced high ROS levels were reduced in OC explants. These results are consistent with previous observations in which HN reduced ROS production, resulting in protection of cells from oxidative stress [10, 21, 26]. In our study, the protective effect of HNG and SHLP3 on hair cells from oxidative stress was also supported by the reduction of gentamicin-induced upregulation of 4-HNE and NRF2 expression. In this context, accumulation of 4-HNE in cochlear tissue exposed to ototoxic drugs induced the expression of NRF2, which in turn reduced the formation of 4-HNE via a glutathione detoxification mechanism [33]. After noise exposure, accumulation of 4-HNE in hair cells was observed in wild-type animals and was even higher in *Nrf2* knockout mice [34]. This suggests that 4-HNE accumulation should be avoided and basal NRF2 expression should be maintained. Therefore, the protective mechanisms of HNG and SHLP3 on hair cells against gentamicin include regulation of ROS production and modulation of 4-HNE and NRF2 expression.

The inflammatory response is a well-known process in gentamicin-induced hair cell damage [8]. We observed that the administration of HNG or SHLP3 reduced gentamicin-induced high levels of IL-1β and IL-6 to control levels. These results are consistent with a previous report showing that HNG attenuates cerebral infarction via inhibition of the expression of the cytokines TNFα, IL-6, and IL-1β [26]. Unfortunately, we did not observe any changes in TNFα transcripts, which could have biological or technical reasons. We hypothesize that in stressful situations, MDPs dampen the production of inflammatory cytokines to alleviate cell damage. However, under non-toxic conditions, the exogenous peptides will work differently to maintain cell health. The increased expression of IL-1b and IL-6 after prolonged treatment with HNG or SHLP3 could indicate that the peptides induced intrinsic effects independent of gentamicin by binding to surface receptors such as the cytokine receptor complex, FPRL receptors, or as yet unknown receptors [17, 18]. The induced inflammation of the peptides could also be a self-regulation of the cells to control the production of RATTIN. In this context, a link between chronic inflammation and humanin regulation in children with inflammatory bowel disease (IBD) was recently reported. The authors found that humanin was downregulated in cultured human growth plates after addition of IBD serum or TNF [35]. Titration of peptide concentrations could be considered to avoid adverse effects of induced inflammation after 40 h, even if no toxic effect on hair cell survival was observed. This is an important aspect to consider in in-vivo studies on the pharmacokinetics and metabolism of MDPs in the inner ear. We can assume that HNG and SHLP3 act against gentamicin-induced inflammation by regulating the expression of inflammatory cytokines.

In summary, we show that exogenous HNG and SHLP3 attenuate gentamicin-induced apoptosis of hair cells by regulating ROS production, controlling the inflammatory response, and modulating the activation of survival proteins, AKT and AMPKα. Our results provide new important insights into the protective mechanism of these bioactive peptides, HNG and SHLP3, under ototoxic conditions. Furthermore, these results open up the possibility of conducting further studies that could promote their use as potential therapeutic peptides to protect hair cells from ototoxicity.

## Material and methods

### Animals

All animal experiments were carried out in accordance with the guidelines and regulations approved by the Animal Welfare Committee of the Canton of Basel, Switzerland (Nr. 2263-35352). The authors complied with the ARRIVE guidelines. Wistar rats were purchased from Janvier Labs (Le Genest-Saint-Isle, France).

### Organ of Corti (OC) explant dissection and experiments

Cochleae were dissected from 4- to 5-day-old rats and incubated as previously described [36]. Briefly, explants were cultured in poly-d-lysine (Sigma Aldrich Chemie GmbH, Steinheim, Germany) coated 8-well Ibidi μ-slides chambers (Vitaris AG, Baar, Switzerland) with 300 µl DMEM/ F12 growth medium (Thermo Fisher Scientific, Reinach, Switzerland), 1× B-27 supplement (Thermo Fisher Scientific), 1× N-2 supplement (Thermo Fisher Scientific) and penicillin (30 U/ml, Sigma Aldrich Chemie GmbH) at 37 °C and 5% CO_2_ for at least 2 h. This was followed by overnight pretreatment with HNG or SHLP3 (Eurogentec S. A., Seraing, Belgium). Then, explants were exposed to gentamicin with or without peptide for 24 h to assess hair cell damage and protein expression, or for 20 h for ROS assay, immunofluorescence, and peptide localization, or for 6 and 16 h to assess RNA expression. OC explants were randomly assigned to different experimental groups.

### Phalloidin staining of cochlear hair cells

The hair cells were stained with Alexa Fluor 568 Phalloidin as previously described [36]. Briefly, at the end of the treatments, the explants were washed with 1× PBS and fixed with 4% paraformaldehyde (PFA, Thermo Fisher Scientific) for 15 min at room temperature. After washing the explants with 1× PBS, the explants were permeabilized with 5% Triton X-100 and 10% FBS in 1× PBS for 15 min at room temperature. Subsequently, hair cells were stained with Alexa Fluor 568 Phalloidin (Thermo Fisher Scientific) at a dilution of 1:150 for 1 h at room temperature in the dark. After staining, Vectashield mounting medium (Reactolab, Servion, Switzerland) was applied to the explants.

### Hair cell counting

Phalloidin-labeled hair cells were visualized using a Nikon Eclipse Ti microscope, equipped with a Yokogawa CSU-W1 spinning, confocal unit (Nikon AG Instruments, Egg, Switzerland), and a Photometrics Prime 95B camera, using NIS (version 5.21). Images were acquired with a 20x air objective (CFI Plan Apo Lambda DM, NA 0.75) as Z stacks of 0.9 μm, which consist of automatically stitched 4 × 4 tiles. Dye was excited using 561 nm laser and ET525/50m emission filter. The number of surviving hair cells was counted using a semi-automated deep learning method integrated into Fiji according to the previously described procedure [37]. Each section to be counted corresponded to a length of 20 inner hair cells. Four sections each were randomly selected for basal, medial and apical cochlear turns. Sections with mechanical damage caused during the experimental procedures were excluded from the analysis. Both inner and outer hair cells were counted separately. Counting results are presented as the number of surviving hair cells per cochlear turn.

### Caspase 3/7 assay

Following treatment, the explants were labeled with 2.5 μM CellEvent Caspase-3/7 Green Detection Reagent (Thermo Fisher Scientific) for 30 min at 37 °C in the dark in complete medium. Subsequently, the organs were fixed with 4% PFA for 15 min at room temperature. The explants underwent additional staining with Alexa Fluor 568 Phalloidin (Thermo Fisher Scientific). Images were acquired using the Nikon Eclipse Ti microscope mentioned above. QuPath (ver 0.4.3) [38] was used for cell segmentation based on nuclear channel Green using StarDist2D plugin [39] with the following parameters: probability threshold: 0.6, pixel size: 0.5 and cell expansion: 0.0 using the model “dsb2018_heavy_augment.pb”, and added intensity measurements. Cells with low GFP mean intensity were excluded. Hair cells were counted in a section corresponding to a length of 20 inner hair cells in three different, randomly selected regions for the basal and middle cochlear turns. The results are presented as the number of positive hair cells per cochlear turn.

### ROS assay

After treatment, the culture medium was replaced by a DCFH-DA solution (ROS Assay Kit-Highly Sensitive DCFH-DA; Dojindo Laboratories, Kumamoto, Japan) and incubated for 30 min at 37 °C and 5% CO_2_. Then the DCFH-DA solution was replaced by a 1× Live Cell Imaging solution (Thermo Fisher Scientific). Images were captured using the above- mentioned Nikon Eclipse Ti microscope within 2 h to determine the production of ROS. QuPath (ver 0.4.3) [38] was used to measure immunofluorescence intensity within a selected section. Each section corresponded to a length of 20 inner hair cells and included inner and outer hair cells. Three sections were randomly selected within the middle cochlear turn. The results are presented as relative DCFH-DA intensity.

### Immunofluorescence

Once treated, the explants were fixed with 4% PFA, permeabilized with 2.5% Triton X-100 for 15 min, and blocked with 0.2% Triton X-100 and 5% goat serum dissolved in 1× PBS for 1 h. The explants were incubated overnight at 4 °C with a primary antibody, followed by incubation with the labeled secondary antibody for 1 h at room temperature. For sequential double labeling, the explants were incubated with the second primary antibody MYO7A overnight at 4 °C, followed by incubation with the second secondary antibody for 1 h at room temperature. Finally, the organs were stained with DAPI for 5 min. The omission of the primary antibody served as a negative control. After staining, the Vectashield mounting medium was carefully applied to each explant. Images were captured using above-mentioned the Nikon Eclipse Ti microscope. QuPath (ver 0.4.3) [38] was used to measure immunofluorescence intensity within a selected section consisting of z-stacks encompassing the entire hair cell bodies. Each section corresponded to a length of 20 inner hair cells and encompassed the inner and outer hair cells as well as the upper part of the supporting cells. Three sections each were randomly selected within the basal and middle turn of the cochlea. Sections showing mechanical damage caused during dissection were excluded from the analysis. The results were presented as intensity measurements.

The following primary antibodies were used: Phospho-AKT (p-AKT)-S473 (1:200, #4060, Cell Signaling, Bioconcept, Allschwil, Switzerland), NRF2 (bs-1074, 1:200, Bioss Inc, Lucerna-Chem AG, Luzern, Switzerland), Phospho-AMPK1/2α (p-AMPKα)-T183/172 (1:200, STJ90735, St John’s Laboratory, Lucerna-Chem AG). The corresponding secondary antibodies were Alexa Fluor 488 goat anti-rabbit IgG and Alexa Fluor 568 goat anti-mouse IgG (Thermo Fisher Scientific).

### Localization of peptides

The explants were pretreated overnight at 37 °C and 5% CO_2_ with FITC-labeled peptides (GenScript Biotech, Rijswijk, Netherlands) and then treated with gentamicin and labeled peptides. After 20 h, the explants were fixed and stained with Alexa Fluor Plus 568 Phalloidin (Thermo Fisher Scientific). Images were acquired using a Nikon Ti2 microscope equipped with AxR laser confocal module with 20x air objective (Plan Apo λ 20x, NA 0.75) (Nikon AG Instruments). Three-channel imaging was done with 405 nm laser for DAPI (MA (PMT), with emission filter range 429–474 nm), 488 nm laser for GFP (GaAsP (PMT), with emission filter range 499–542 nm), and 561 nm laser for Cy3 (GaAsP (PMT), with emission filter range 571–625 nm), with a pinhole size 19.6 μm. Galvano Unidirectional mode with 2x averaging was activated. Z stack of around 30 μm with a Z step of 0.7 μm was acquired. The final image was deconvolved using Nikon NIS (ver. 5.40.02).

Intensity line profiles were created using the Intensity Profile tool of the NIS software. For each image, a line of 100 µm length was drawn in a single XY plane. The line was drawn randomly in the middle region. The diagram shows the distribution of pixel intensities along the defined linear section.

### Western Blot analysis

After treatment, explants were washed with 1× PBS and transferred into the T-PER lysis buffer (Thermo Fisher Scientific) containing protease and phosphatase inhibitors (Roche Applied Science, Rotkreuz, Switzerland). Next, the protein concentration was measured using a BCA protein assay (Thermo Fisher Scientific) following the instructions of the manufacturer. The samples were then diluted to equal concentrations and volumes. The proteins were separated by electrophoresis and transferred to PVDF membranes. Immunoblotting was performed with iBind Automated Western Systems (Thermo Fisher Scientific) following the instructions of the manufacturer. West Femto Super Signal kit (Thermo Fisher Scientific) was used for chemiluminescence detection of the antibodies. Immunoblots were always analyzed first for phosphorylated forms of proteins, stripped with Restore PLUS Western Blot Stripping Buffer (Thermo Fisher Scientific), and re-probed with the corresponding antibodies for total proteins. β-Actin was used as a protein loading control. At least six explants were used for each condition.

Primary antibodies were used at the following dilutions: Phospho-STAT3 (p-STAT3)-Y705 at 1:2000 (#9131), Phospho-AMPKα (p-AMPKα)-T172 at 1:500 (#2535), AMPKα at 1:1000 (#5832), BAX at 1:1000 (#14796), ERK1/2-T202/Y204 at 1:1000, (#4370) Phospho-AKT (p-AKT)-S473 at 1:2000 (#4060), AKT at 1:1000 (#9272; all from Cell Signaling). STAT3 at 1:1000 (sc-482, Santa Cruz Biotechnology, LabForce AG, Muttenz, Switzerland), NRF2 at 1:1000 (bs-1074, Bioss Inc), Rattin at 1:500 (NB300-246, Novus Biological, Zug, Switzerland), Anti-4-Hydroxynonenal (4-HNE, ab46545) at 1:1000, β-actin at 1:5000 (ab3280, Abcam, Labforce AG, Nunningen, Switzerland).

### Quantitative real-time PCR

A pool of four to five OC explants was used for each group. The cultured organs were stored in RNA Later (Qiagen, Hombrechtikon, Switzerland) at 4 °C until use. RNA isolation was performed using the Quick-DNA/RNA MagBead Kit (Zymo Research, CA, USA) according to the manufacturer’s instructions. First strand cDNA was synthesized using the GoScript Reverse Transcription Mix Kit (Promega, Dübendorf, Switzerland). Real-time PCR was performed using the GoTaq qPCR Master Mix Kit (Promega). All primers used in this study were purchased from Microsynth (Mycrosynth AG, Balgach, Switzerland). The primers were obtained from previous studies or were self-designed using Primers3 and BLAST (NCBI) and are listed in Table S1.

Each sample was run in triplicate in the ViiA 7 Real-time PCR system (Thermo Fisher Scientific). The reactions were initially denatured at 95 °C for 2 min, followed by 40 cycles at 95 °C for 3 s and 60 °C for 30 s. The relative transcript levels were calculated using the 2^−ΔΔCT^ method. *Hprt*, *Actb*, and *Gapdh* were selected as the reference genes.

### Statistical analysis

All values obtained in the present study are presented as mean+S.D. Statistically analysis was performed using GraphPad Prism 9 software. The two-way ANOVA method was used to analyze the differences among groups. *P* < 0.05 was considered statistically significant.

## Supplementary information

FITC-HNG

FITC-SHLP3

Video legends

SUPPLEMENTAL FILES

ORIGINAL WB

## Data Availability

The data supporting the results of this study are available on reasonable request from the corresponding author.
