# Supplementary material for: Mitochondrial-derived peptides, HNG and SHLP3, protect cochlear hair cells against gentamicin
Source: Cell Death Discov. 2024 Oct 21;10:445. doi: 10.1038/s41420-024-02215-9 (PMC11493991; doi:10.1038/s41420-024-02215-9)
Supplement: Supplementary file 3 — Video legends [file 41420_2024_2215_MOESM3_ESM.docx]

**Mitochondrial-derived peptides, HNG and SHLP3, protect cochlear hair cells against gentamicin**

Yu Lu^1^; Ewelina M. Bartoszek^1^; Maurizio Cortada^1,2^; Daniel Bodmer^1,2^; Soledad Levano^1*^

^1^Department of Biomedicine and ^2^Department of Otolaryngology, Head and Neck Surgery, University of Basel Hospital, Basel, Switzerland

**Supplementary Video 1.**

**Detection of exogenous FITC-HNG in a section of the middle turn of the cochlea.** The hair cells were stained with phalloidin (orange) and nuclei with DAPI (blue). For better visualization, the brightness of the images was enhanced using the LUTs tools of the NIS software.

**Supplementary Video 2.**

**Detection of exogenous FITC-SHLP3 in a section of the middle turn of the cochlea.** The hair cells were stained with phalloidin (orange) and nuclei with DAPI (blue). For better visualization, the brightness of the images was enhanced using the LUTs tools of the NIS software.
