# Supplementary material for: Mitochondrial-derived peptides, HNG and SHLP3, protect cochlear hair cells against gentamicin
Source: Cell Death Discov. 2024 Oct 21;10:445. doi: 10.1038/s41420-024-02215-9 (PMC11493991; doi:10.1038/s41420-024-02215-9)
Supplement: Supplementary file 4 — SUPPLEMENTAL FILES [file 41420_2024_2215_MOESM4_ESM.docx]

**Mitochondrial-derived peptides, HNG and SHLP3, protect cochlear hair cells against gentamicin**

Yu Lu^1^; Ewelina M. Bartoszek^1^; Maurizio Cortada^1,2^; Daniel Bodmer^1,2^; Soledad Levano^1*^

^1^Department of Biomedicine and ^2^Department of Otolaryngology, Head and Neck Surgery, University of Basel Hospital, Basel, Switzerland

Corresponding author:

Dr. Soledad Levano

Department of Biomedicine

University of Basel Hospital

4031 Basel

Switzerland

Phone: +41 61 265 23 97

Email: [S.levano@unibas.ch](mailto:S.levano@unibas.ch)

**Table S1. Primers sequences used for qPCR.**

| **Gene** | **Primer sequence** |
| --- | --- |
| F-IL-1b | 5’- AACTGTGAAATAGCAGCTTTCG -3' |
| R-IL-1b | 5’- CTGTGAGATTTGAAGCTGGATG -3' |
| F-IL6 | 5’- GCCCACCAGGAACGAAAGTC -3' |
| R-IL6 | 5’- TGGCTGGAAGTCTCTTGCG -3' |
| F-TNFa | 5’- ATGGGCTCCCTCTCATCAGT -3' |
| R-TNFa | 5’- GCTTGGTGGTTTGCTACGAC -3' |
| F-HPRT | 5’- CTCATGGACTGATTATGGACAGGAC -3' |
| R-HPRT | 5’- GCAGGTCAGCAAAGAACTTATAGCC -3' |
| F-Actin | 5’- CCCTGGCTCCTAGCACCAT -3' |
| R-Actin | 5’- AGAGCCACCAATCCACACAGA -3' |
| F-GAPDH | 5’- GCATCTTCTTGTGCAGTGCC -3' |
| R-GAPDH | 5’- TACGGCCAAATCCGTTCACA -3' |

**Supplementary Figure S1.**

**Additional tested concentrations of SHLP3.** Quantification of inner hair cell (IHC) and outer hair cell (OHC) survival in the basal, middle and apical regions at different concentrations of SHLP3 between 0.04 and 0.4 µM in OC explants. At least three explants for each condition were used. Values are presented as mean + D. ****P;<0.0001, ns not significant.

**
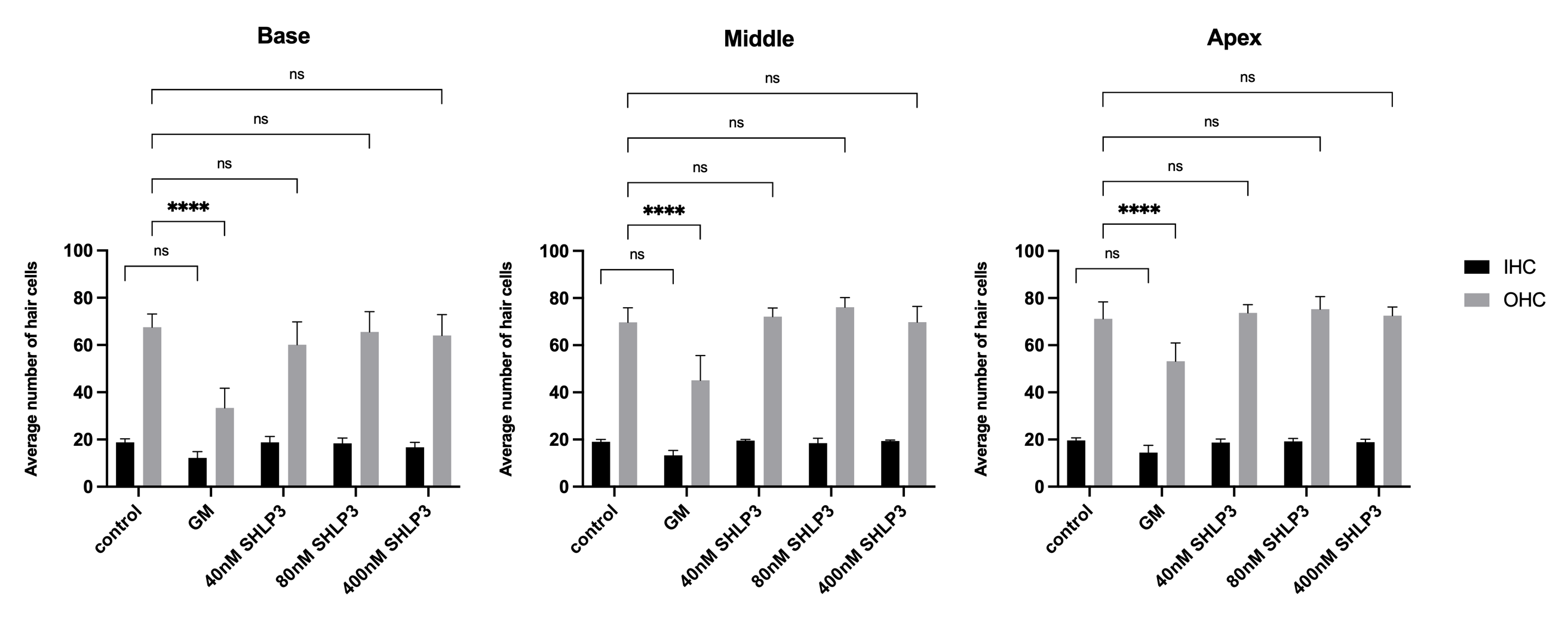
**

**Supplementary Figure S2.**

**Exogenous HNG and SHLP3 are taken up into cochlear cells when exposed to gentamicin.** A) Schematic representation of the cross-section of the cochlear organ with inner hair cells (IHC), outer hair cells (OHC) and supporting cells such as inner border cells (IBC), inner phalangeal cells (IPhCs), pillar cells (PCs), Deiters’ cells (DCs), and Hensen’s cells (HeCs). B) Representative images of FITC-HNG (green) and FITC-SHLP3 (green) in the middle regions of the cochlea from a YZ-projection image. The left images are the composite images and the right images show only the FITC fluorescence. To better visualize the peptides, different fluorescence intensities were used for FITC-HNG and FITC-SHLP3, while maintaining the settings within each peptide group. Solid white lines indicate the IHC, OHC, and support cells. Hair cells (orange) and nuclei (blue) were stained with phalloidin and DAPI, respectively. n = 4. Scale bar = 10 µm.

**
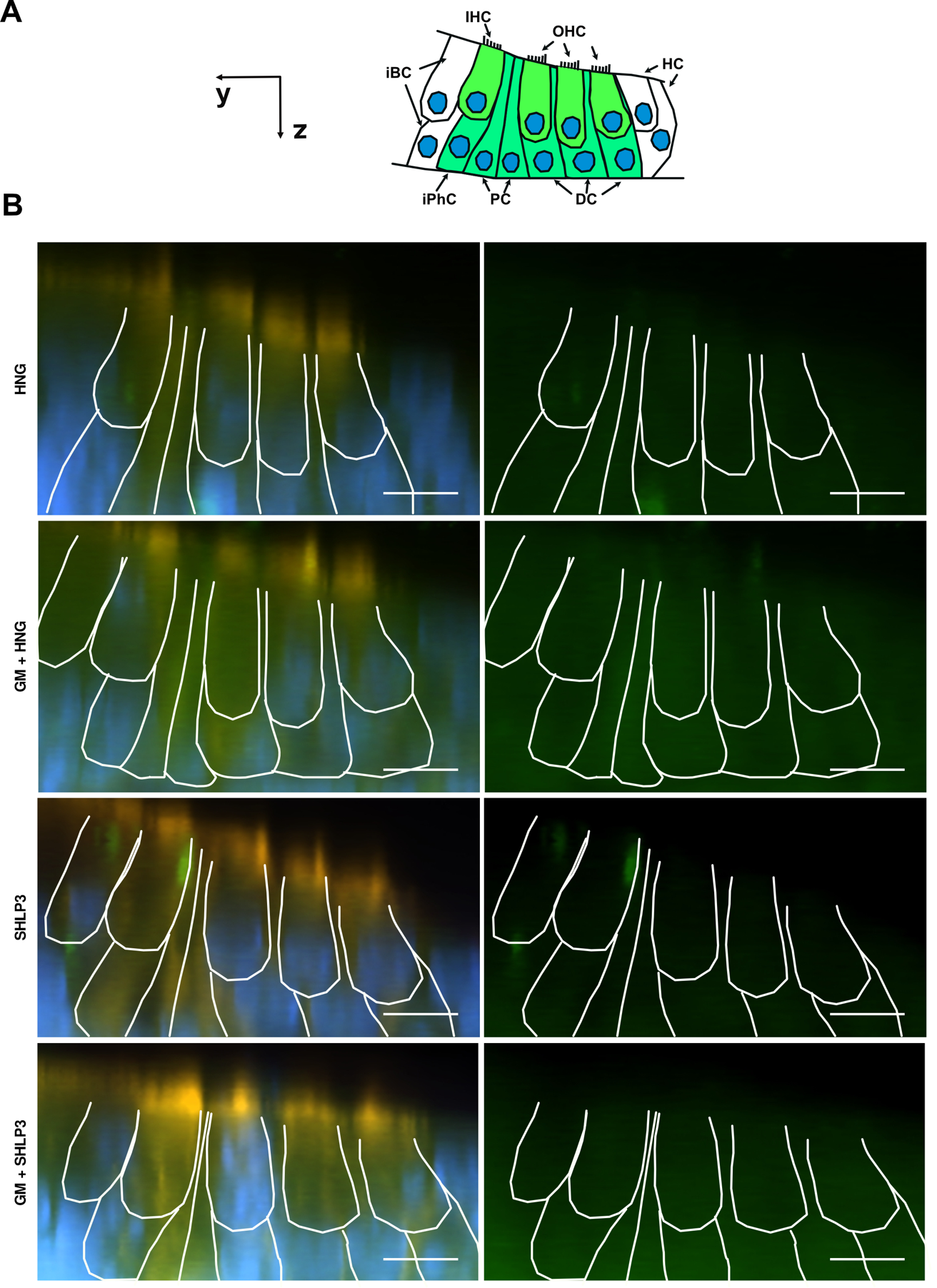
**
