# Supplementary material for: Mitochondrial-derived peptides, HNG and SHLP3, protect cochlear hair cells against gentamicin
Source: Cell Death Discov. 2024 Oct 21;10:445. doi: 10.1038/s41420-024-02215-9 (PMC11493991; doi:10.1038/s41420-024-02215-9)
Supplement: Supplementary file 5 — ORIGINAL WB [file 41420_2024_2215_MOESM5_ESM.pdf]

Figure S3

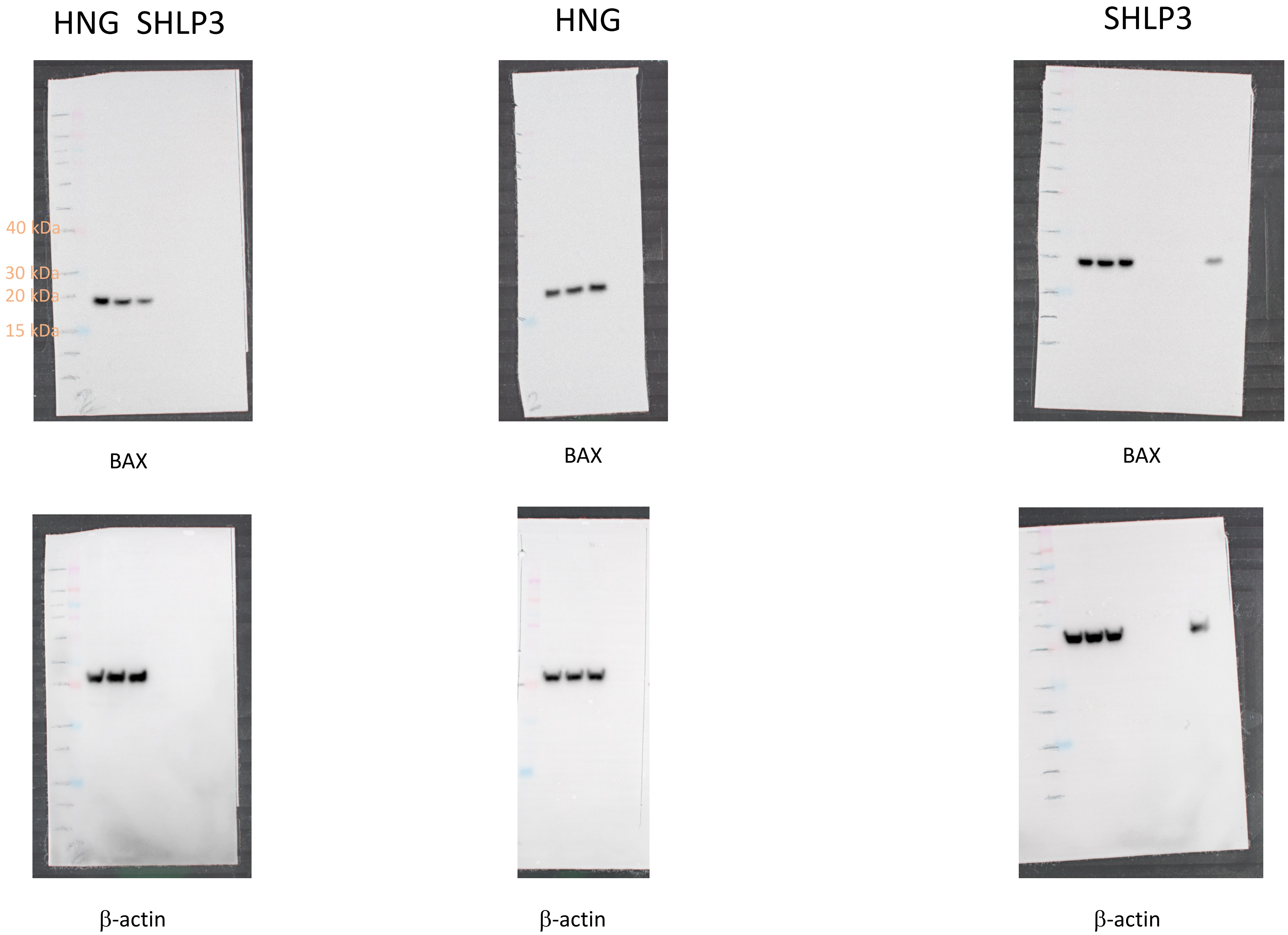
HNG SHLP3
HNG
SHLP3
40 kDa
30 kDa
20 kDa
15 kDa
BAX
BAX
BAX
β-actin
β-actin
β-actin

Figure S4

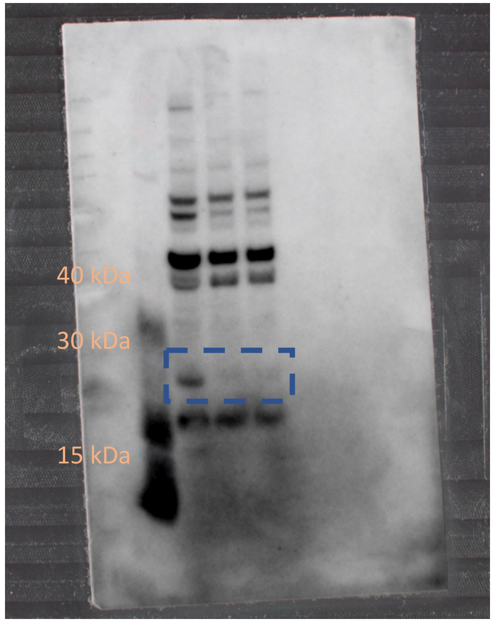

Rattin (Novus biologicals), predicted 25 kDa

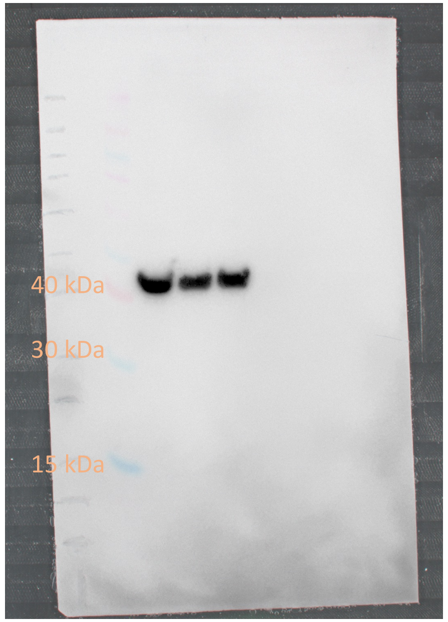

β-actin

Figure S5

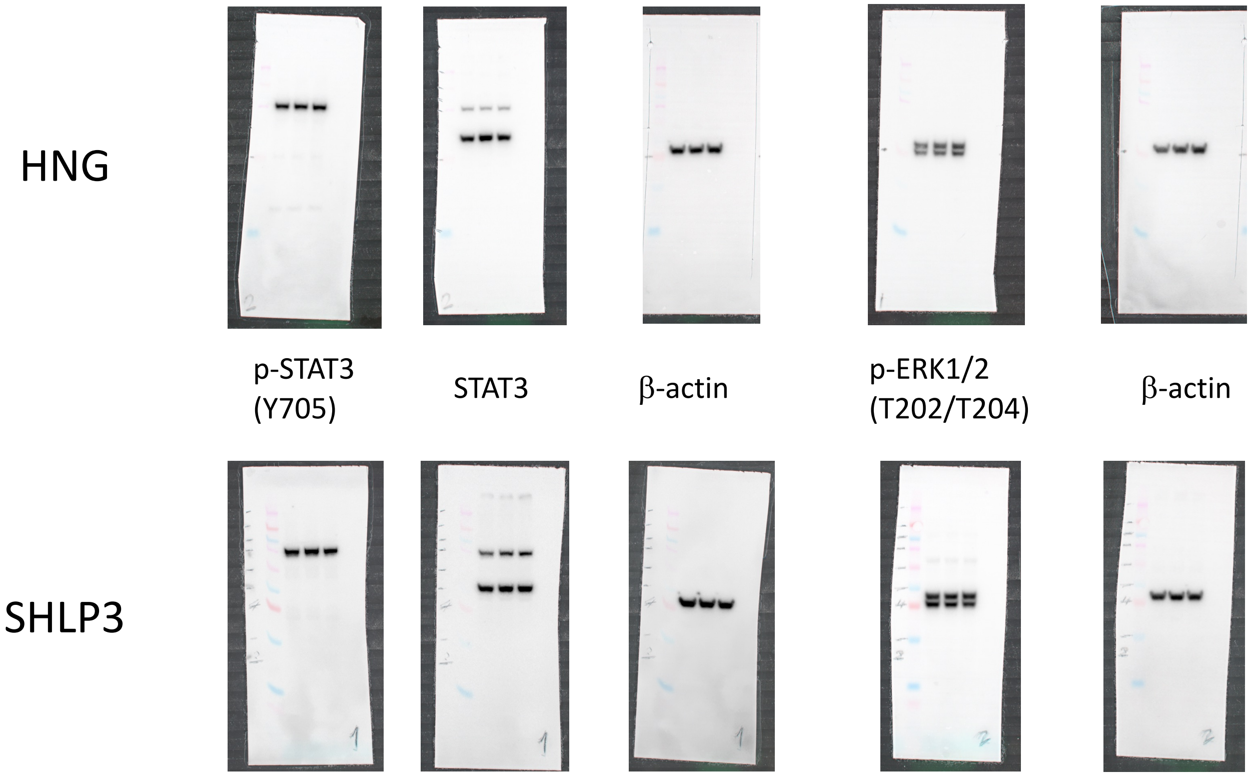

Figure S6

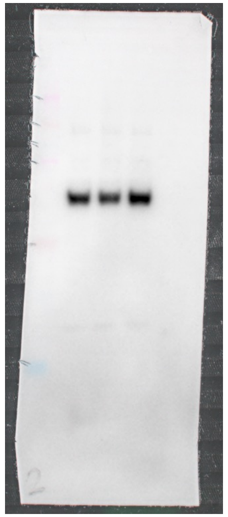

p-AKT
(S473)

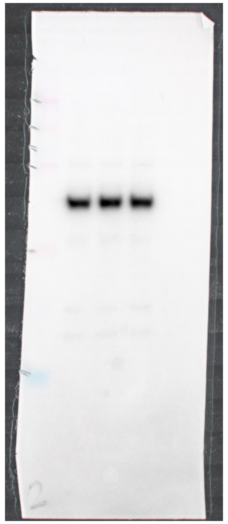

AKT

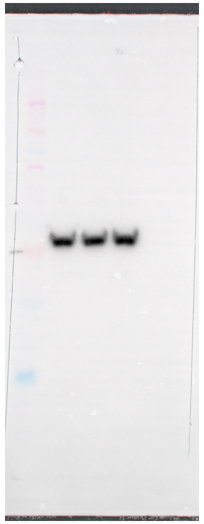

β-actin

Figure S7

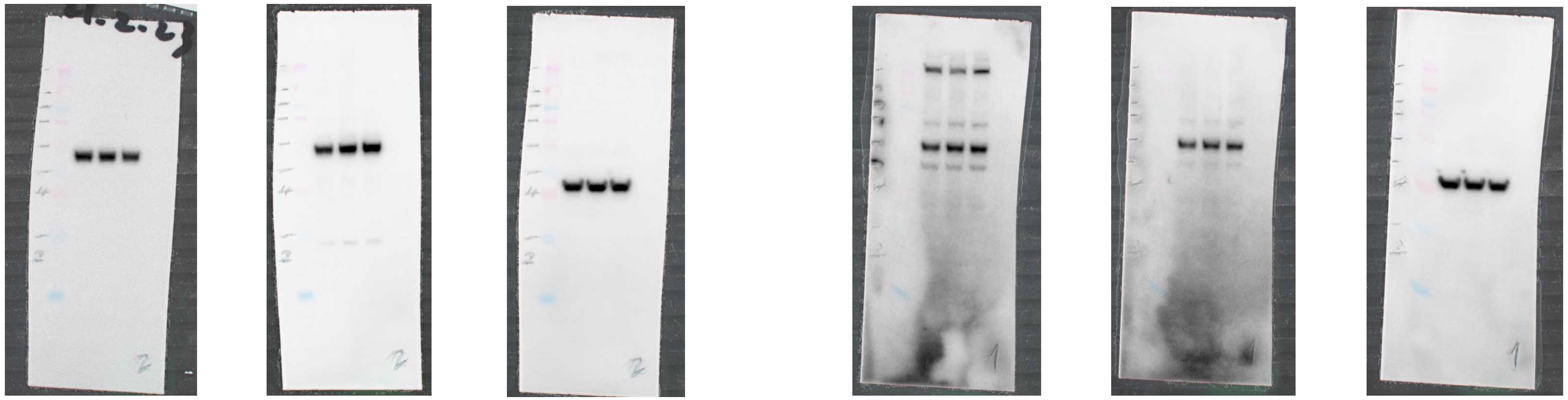

p-AKT (S473) | AKT | β-actin | p-AMPKα (T172) | AMPKα | β-actin

Figure S8

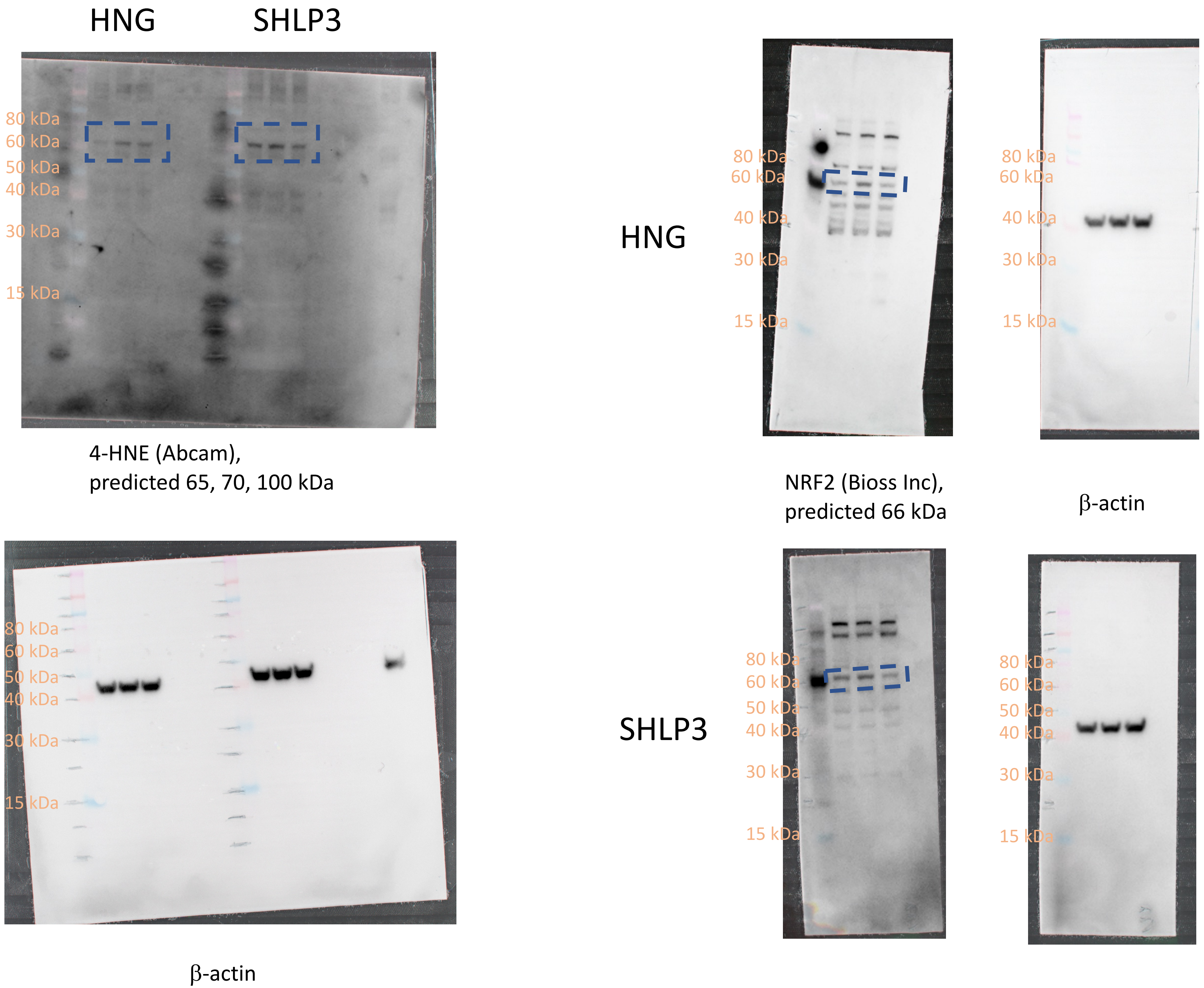
HNG
SHLP3
80 kDa
60 kDa
50 kDa
40 kDa
30 kDa
15 kDa
4-HNE (Abcam),
predicted 65, 70, 100 kDa
β-actin
HNG
SHLP3
NRF2 (Bioss Inc),
predicted 66 kDa
β-actin
